# Global Genomic Characterization of *Salmonella enterica* Serovar Telelkebir

**DOI:** 10.3389/fmicb.2021.704152

**Published:** 2021-07-29

**Authors:** Yu-feng Qiu, Reshma B. Nambiar, Xue-bin Xu, Shun-tai Weng, Hang Pan, Kui-cheng Zheng, Min Yue

**Affiliations:** ^1^Department of Bacterialogy, Fujian Provincial Center for Disease Control & Prevention, Fuzhou, China; ^2^Department of Bacterialogy, Fujian Provincial Key Laboratory of Zoonosis Research, Fuzhou, China; ^3^Department of Veterinary Medicine & Institute of Preventive Veterinary Science, Zhejiang University College of Animal Sciences, Hangzhou, China; ^4^Department of Microbiology, Shanghai Municipal Center for Disease Control and Prevention, Shanghai, China; ^5^School of Public Health, Fujian Medical University, Fuzhou, China; ^6^Zhejiang Provincial Key Laboratory of Preventive Veterinary Medicine, Hangzhou, China; ^7^State Key Laboratory for Diagnosis and Treatment of Infectious Diseases, National Clinical Research Center for Infectious Diseases, National Medical Center for Infectious Diseases, The First Affiliated Hospital, College of Medicine, Zhejiang University, Hangzhou, China; ^8^Hainan Institute of Zhejiang University, Sanya, China

**Keywords:** *Salmonella* Telelkebir, invasive infection, antimicrobial resistance gene, typhoid toxin, virulence gene

## Abstract

Non-typhoidal *Salmonella* (NTS) is a common cause for self-limiting gastroenteritis, representing a public health concern globally. NTS is one of the leading causes of foodborne illnesses in China; however, the invasive infection caused by NTS is largely underappreciated. Here, we reported an NTS invasive infection caused by an infrequently reported serovar Telelkebir (13,23:d:e,n,z15) strain FJ001 in China, which carries antimicrobial-resistant genes [*fosA7* and *aac(6′)-Iaa*] and typhoid-toxin genes (*cdtB*, *pltA*, and *pltB*). By conducting the whole genomic sequencing, we also investigated the relatedness of this strain with an additional 120 global contextual *Salmonella enterica* serovar Telelkebir (*S.* Telelkebir) isolates, and assessed the antimicrobial-resistant determinants and key virulence factors using the available genomic dataset. Notably, all 121 (100%) of the *S.* Telelkebir strains possessed the typhoid toxin genes *cdtB*, *pltA*, and *pltB*, and 58.67% (71/121) of *S*. Telelkebir harbored antimicrobial-resistant gene *fosaA7*. The study by core genome multilocus sequence typing (cgMLST) and core single-nucleotide polymorphism (SNP)-based phylogenomic analysis demonstrated that the *S*. Telelkebir isolates from different sources and locations clustered together. This suggests that regular international travels might increase the likelihood of rapid and extensive transmissions of potentially pathogenic bacteria. For the first time, our study revealed the antimicrobial resistance, virulence patterns, and genetic diversity of the serovar *S.* Telelkebir isolate in humans and similar isolates over the world. The present study also suggests that genomic investigation can facilitate surveillance and could offer added knowledge of a previously unknown threat with the unique combination of virulent and antimicrobial-resistant determinants.

## Introduction

*Salmonella enterica* is a major global foodborne pathogen (Pan et al., 2018; World Health Organization (WHO), 2018; Jiang et al., 2021). *S. enterica* is divided into six distinct subspecies: *enterica*, *salamae*, *arizonae*, *diarizonae*, *houtenae*, and *indica* (Chand et al., 2020). The *S. enterica* subsp. *enterica* consists of more than 1,500 serotypes. A small number of *Salmonella* serovars (*S*. Typhi and *S*. Paratyphi A, B, or C) are human restricted and evoke an invasive, life-threatening systemic disease (Parry et al., 2002; Liu et al., 2021). In contrast, the non-typhoidal *Salmonella* (NTS) serovars generally cause self-limiting diarrheal illnesses with low case mortality. According to the Global Burden of Diseases, Injuries, and Risk Factors Study (GBD 2017 Causes of Death Collaborators, 2018), it was estimated that NTS accounts for 95 million cases of enterocolitis and 50,771 related deaths.

In sub-Saharan Africa, NTS is the most common cause of bloodstream infection in immunocompromised adults and children with a fatality rate of 20–25% (Feasey et al., 2012). In developed countries, 5% of NTS cases are invasive, focal systemic infections, or extra-intestinal disease leading to bacteremia (Suez et al., 2013). The NTS infections in humans arise through the food chain, but infection can also be contracted through contact with the infected animals, person-to-person transmission, or contaminated water (World Health Organization (WHO), 2018). Various wildlife animals, reptiles, and their contaminated environment could act as a reservoir for various rare and infrequently reported serovars that are normally dissimilar from those strains isolated from the commercial food chain. Currently, most of the research is concentrated on *S*. Typhimurium and *S*. Enteritidis, and comparatively lesser consideration has been given to uncommon emerging serovars (Tankson et al., 2006; Mansour et al., 2020). There is an evolving need to investigate the incidence of these uncommon *S. enterica* serovars as a variety of these have been linked to numerous foodborne outbreaks, acute gastroenteritis, splenic abscesses, etc. (Rodriguez et al., 2006; Berendes et al., 2007; Li et al., 2017), which are largely underappreciated. In China, most of the foodborne bacterial outbreaks (70–80%) are ascribed to *Salmonella* infections (Wang et al., 2019; Paudyal et al., 2018; Li et al., 2020; Zhou et al., 2020). However, the study on invasive NTS infection remains incomplete in China.

Recently, various reports have indicated the efficiency of whole-genome sequencing (WGS) in the epidemiological investigation of transmittable disease at different geographical locations (Gardy and Loman, 2018). The WGS methods involve either the characterization of core single-nucleotide polymorphisms (SNP) or core-genome multilocus sequence typing (cgMLST) analysis (Mellmann et al., 2016). The cgMLST-based approach involves the direct assessment and comparison of newly determined genotypes with historically available data. However, the SNP-based analysis requires recalculation once there is a change in the data set unless a preliminarily established reference genome is provided (Ruan et al., 2020).

In this study, we have investigated a bloodstream infection case caused by *Salmonella* Telelkebir in China. To improve our knowledge of the genomic epidemiology of *S*. Telelkebir, phylogenetic relationship and comparative genomic analysis of additional 120 global *S*. Telelkebir isolates from various countries were also carried out.

## Materials and Methods

### Ethics Statements

The FJ001 strain was collected and approved by the Shanghai and Fujian Center for Disease Control and Prevention. Written consent was obtained prior to the study for collecting the samples for surveillance purposes.

### Bacterial Isolates

**FIGURE 1:**
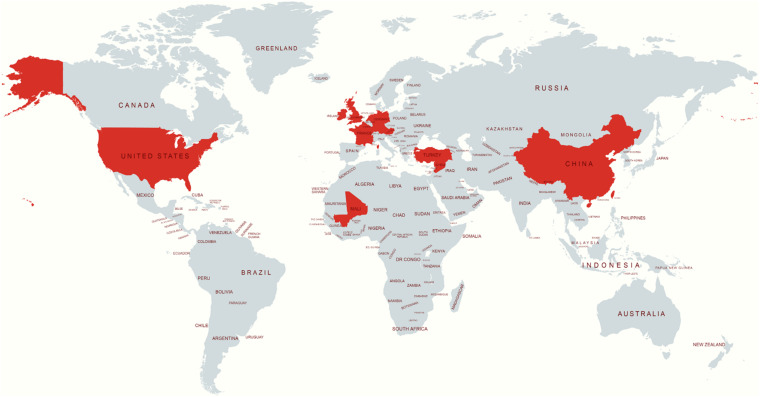
Source location of *S*. Telelkebir strain genomes used in this study. The red color indicates the presence of this serovar. This map was created using an online service (https://mapchart.net/).

The publicly available isolates with their metadata were collected from Enterobase^1^ or the sequence read archive^2^. The *S.* Telelkebir isolates were used in this study to embody various geographical locations, including the UK (*n* = 61), United States (*n* = 23), Germany (*n* = 18), Ireland (*n* = 4), France (*n* = 4), China (*n* = 1), Mali (*n* = 1), Netherlands (*n* = 1), and Turkey (*n* = 1). The locations of the remaining strains were not available (NA) (Figure 1).

### Clinical Study

**FIGURE 2:**
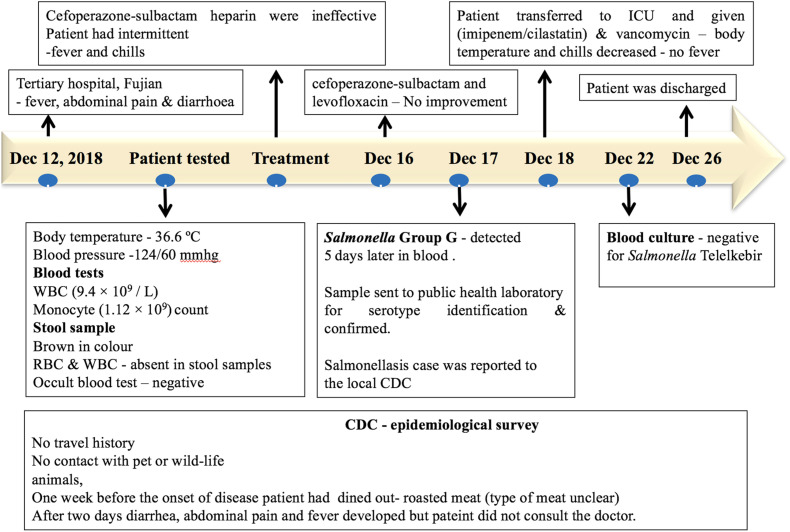
Timeline of the salmonellosis diagnosis, treatment, and investigation since December 12, 2018.

A patient with no significant medical history was admitted to a tertiary hospital for cardiovascular medicine in Fujian, with fever, abdominal pain, diarrhea, and syncope on December 12, 2018. The patient had no history of travel or contact with any wildlife or domestic animals. The patient had a history of eating outside (roasted meat, the type of meat was unclear) 1 week prior to the onset of the disease. However, other food sources may have been the vehicle of infection. During admission, the patient had a body temperature of 36.6°C, and the blood pressure was 124/60 mmHg. Blood tests showed a normal white blood count (9.4 × 10^9^/L) and a monocyte count (1.12 × 10^9^/L). The stool sample was normal and was negative for fungi and parasites *via* microscopic examination. The stool sample was negative for *Salmonella* and *Shigella*. On the second day of hospital admission, the patient developed diarrhea, abdominal pain, and fever. The injection treatments with Sulperazon (cefoperazone sulbactam) and heparin were ineffective. During the treatment, the patient had intermittent fever and chills. On December 16, a combination of treatments with Sulperazon (cefoperazone sulbactam) and Cravit (levofloxacin) injection also failed. On December 17, the bacterial blood culture indicated the presence of *Salmonella* Group G, and the patient was diagnosed for sepsis and multiple organ dysfunction syndromes. On December 18, the patient was transferred to the intensive care unit with combined treatment of imipenem–cilastatin and vancomycin, and the body temperature of the patient and chills decreased. After a successive 4-day treatment, the blood culture was negative for *Salmonella*. The patient completely recovered on December 26 and was discharged from the hospital (Figure 2).

### Characterization of *Salmonella* Telelkebir

For screening *Salmonella* and *Shigella*, blood and stool samples were spread onto xylose lysine deoxycholate agar (XLD) and MacConkey agar, respectively, and were incubated at 36°C for 24 h. The stool cultures were negative for *Salmonella*, while the blood culture displayed positive colonies on XLD after 24 h. The plate was purple-red in color, and colonies with the typical characteristic of *Salmonella*, i.e., round, moist, smooth, colorless, translucent, and black in the center were selected and inoculated in triple sugar iron (TSI) plate for 24 h at 36°C. The *Salmonella* isolate was further subjected to biochemical analysis (VITEK2 COMPACT; bioMérieux, France) and identified using matrix-assisted laser desorption/ionization time-of-flight mass spectrometry (MALDI-TOF-MS). The serotype agglutination assay was conducted using the anti-serum purchased from Denmark (SSI Diagnostica, Denmark), and was confirmed as a newly reported serovar (13,23:d:e,n,z15 or Telelkebir) in mainland China.

### Antimicrobial Susceptibility Testing

The bacteria isolate was used for antimicrobial susceptibility testing (AST) against 17 antimicrobial agents. Broth microdilution minimum inhibitory concentration (MIC) determination was performed according to the Clinical and Laboratory Standards Institute (CLSI) guidelines and interpretation (CLSI, 2018), the European Committee for Antimicrobial Susceptibility Testing (EUCAST, 2018), and where no EUCAST or CLSI interpretative criteria were available, breakpoints were harmonized with those of the National Antimicrobial Resistance Monitoring System (NARMS), United States (FDA, 2017). The following antimicrobials were tested: fosfomycin, levofloxacin, cefoperazone sulbactam, streptomycin, ampicillin, amoxicillin–clavulanic acid, cefoxitin, imipenem, nalidixic acid, ciprofloxacin, chloramphenicol, tetracycline, kanamycin, gentamicin, trimethoprim–sulfamethoxazole, ceftiofur, and azithromycin (Sangon Biotech, China). *Escherichia coli* ATCC 25922 were used as a control strain. The AST against all antibiotics were carried out in Muller–Hinton broth (MHB) medium in both aerobic and anaerobic conditions and incubated at 37°C. The AST was also performed in Dulbecco’s modified Eagle’s medium (DMEM) (Dulbecco and Freeman, 1959) and incubated in a 5% CO_2_ incubator.

### DNA Extraction, Genomic Sequencing, and Data Analysis

The genomic DNA of FJ001 isolate was extracted from overnight cultures grown at 37°C in Luria–Bertani broth under 180 rpm shaking conditions by using a TIANamp bacteria DNA kit (Tiangen Biotech, China) according to the manufacturer’s protocol. The Qubit Broad Range assay kit (Invitrogen, United States) was used for quantification. The Genomic DNA library was constructed using Nextera XT DNA library construction kit (No. FC-131-1024; Illumina, United States). High-throughput genome sequencing was accomplished by the Illumina NovaSeq 6000 platform, using paired-end sequencing of 2 × 150-bp reads as previously described (Biswas et al., 2019; Paudyal et al., 2019; Yu et al., 2020). For the comparative genomic analysis, an additional 119 isolates (Supplementary Table 1), with assembled contigs in FASTA format, were obtained from the Enterobase (see text footnote 1). One additional strain, with raw reads in SRA format was obtained from the NCBI SRA dataset (see text footnote 2). The quality of sequencing was checked with FastQC toolkit (Rouzeau-Szynalski et al., 2019). The raw reads of the FJ001 genome were trimmed with trimmomatic software prior to genome assembly. The raw reads for each strain were assembled by using SPAdes 4.0.1 (Bolger et al., 2014). QUAST (Gurevich et al., 2013) was used to assess the assembled genomes through basic statistics generation, including the total number of contig, the length of contig, and N50 (Supplementary Table 2). Prokka v.1.14 with default settings under the in-house galaxy platform and Rapid Annotation Subsystem Technology (RAST) server^3^ were used for annotation of the assembled genomes. The NCBI Basic Local Alignment Search Tool (BLAST) BLASTp^4^ program was used for similarity alignment. The plasmid types and antimicrobial-resistant genes were determined using the PlasmidFinder 2.0^5^ and ResFinder 3.2,^6^ respectively (Zankari et al., 2012). The virulence factors in the genome were examined using the Virulence Factors Database (VFDB) (Liu et al., 2019). *Salmonella* Telelkebir *in silico* serotyping was conducted by the *Salmonella In Silico* Typing Resource (SISTR) platform^7^ and SeqSero2.^8^ The multilocus sequence typing of the isolates were carried out using MLST.^9^ The contigs were used for variant calling against reference strain 98-12414 and outgroup control strain Poona ATCC^®^ BAA-1673 by software Snippy v4.4.4 to obtain core single-nucleotide polymorphism (SNP) for determining the population structure of 121 available *S*. Telelkebir isolates. After being filtered by 95% gap parameter to get the core SNPs, a total of 85,694 SNPs were used to build a maximum-likelihood phylogenetic tree with 1,000 bootstraps using IQ-TREE v.1.6.12 with the best model TVM + F + ASC (Letunic and Bork, 2007).

### Core Genome Multilocus Sequence Typing Analysis of *Salmonella* Telelkebir Isolates

The cgMLST analysis was carried out using the Ridom SeqSphere+ software v6.0.2 (Ridom). An *ad hoc* core genome MLST (cgMLST) scheme was created for the gene-by-gene analysis with SeqSphere+ (Ridom^®^ GmbH, Münster, Germany). Hence, the *S*. Typhimurium LT2 (NC_003197.1) genome comprising 4,451 genes was used as annotated reference. The cgMLST target definer tool was applied to 121 *Salmonella* Telelkebir genomes with the default settings of the software to define the core genome loci (Simon et al., 2018). A cgMLST tree was built using the neighbor-joining method. The cgMLST distance matrix showing pairwise comparison of allelic differences between 121 isolates is given in Supplementary Table 3.

### Data Availability

The genome for the strain FJ001 was deposited in the NCBI (BioProject PRJNA666303). Associated metadata and virulence genes can be found in Supplementary Table 1.

## Results

### *Salmonella* Telelkebir Clinical Isolate FJ001

In this study, we reported a bloodstream infection caused by *Salmonella* Telelkebir (FJ001) isolate in mainland China. The *Salmonella* isolate was serotyped by agglutination assay at both the Fujian and Shanghai Center for Disease Prevention and Control, and two independent groups confirmed this causative isolate as an uncommon serovar *S. enterica* subsp. *enterica* Telelkebir (13,23:d:e,n,z15). Here, a retrospective investigation confirmed that the patient had no history of travel and pet contact for 1 month prior to the onset of the disease. Exposure factors are mainly related to the history of unclean diet, i.e., roasted meat, but this cannot make accurate traceability judgments for the source of infection. Also, other food source may have been the vehicle of infection. However, Fujian province, located in the sub-tropic region of Eastern China, has abundant natural species resources, local processing, and consumption habits of wild and farmed snakes. Therefore, we cannot rule out the possibility of infection sources as snakes and other reptiles (Yu-feng et al., 2020), for this particular case.

### Antimicrobial Resistance Profile for FJ001

The MIC analysis showed that the strain FJ001 was susceptible against all examined antimicrobial agents in both the tested medium under the tested conditions (Supplementary Table 4). It is interesting to note that earlier treatment with cefoperazone sulbactam alone or combined with levofloxacin failed to inhibit the bacterial replication in the patient.

### Antimicrobial Resistance Genes and Plasmids in *Salmonella* Telelkebir Population

**FIGURE 3:**
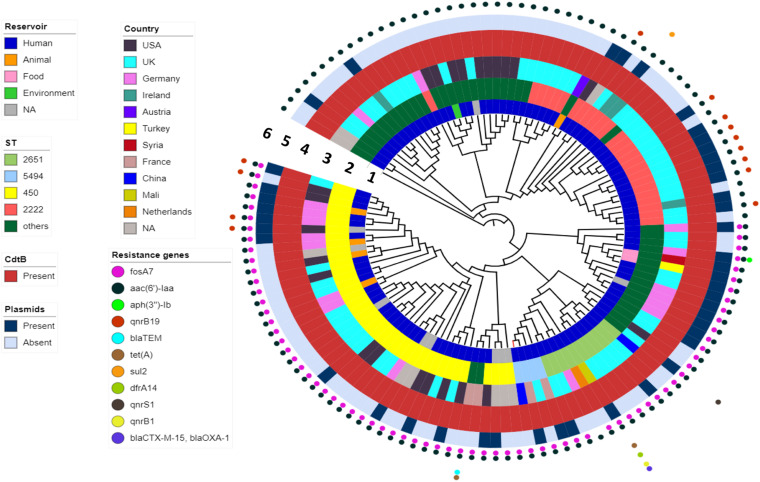
The phylogenomic relationship among 121 *Salmonella* Telelkebir strains. The tree has been rooted using the serovar Poona ATCC^®^ BAA-1673 as the outgroup. The numbers 1–6 represents the inner to outer rings corresponding to reservoir, ST, country, *cdtB*, plasmids, and resistance genes, respectively. The strain FJ001 containing leaf node is represented in red. The following plasmids were present in the *Telelkebir* strains: IncI1α, IncFII, Col440I, IncHI2, IncN, and IncI2 (Delta).

WGS analysis indicated that the FJ001 strain carries two antimicrobial-resistant genes, *fosA7* and *aac(6′)-Iaa*. To understand the global resistance patterns, plasmid profile, and virulence patterns of the *S*. Telelkebir strains, an additional 120 strains were retrieved from Enterobase and NCBI. The WGS analysis revealed that the global strains contains resistance genes against eight antibiotic families: aminoglycosides including *aac(3)-Vla* (1/121), *aac(6′)-Iaa_1* (121/121), *ant(3″)-Ia_1* (1/121), *aph(3)-Ib_5* (2/121), and *aph(6)-Id_1* (3/121); beta-lactamases, including *bla*_TEM–1B_ (3/121), *bla*_CTX–M–15_ (1/121), and *bla*_OXA–__1_ (1/121); phenicols, including *catB3_1* (1/121) and *catA1_1* (1/121); trimethoprims including *dfrA14_5* (1/121); quinolones including *qnrB19_1* (10/121), *qnrB1_1* (2/121), and *qnrS1_1* (1/121); sulfonamides, including *sul1_5* (12/121) and *sul2_2* (2/121); tetracyclines, including *tet(A)* (3/121), and fosfomycins including *fosA7_1* (71/121) (Figure 3). All the *S.* Telelkebir genomes harbored at least one antimicrobial-resistant gene *aac(6′)-Iaa_1* (Supplementary Table 1). A total of 58.67% (71/121) isolates harbored resistance genes for fosfomycin, and 11.57% (14/121) isolates possessed resistance gene for quinolones. Three isolates recovered from human infections from different countries harbored antimicrobial resistance gene *bla*_TEM–1B_. Two human isolates from Mali (07-1331) and UK (56980) harbored resistance genes to more than three antibiotic families (Figure 3), which indicates that these strains could be multiple drug-resistant (Elbediwi et al., 2019; Mansour et al., 2020). A total of 35.2% isolates harbored plasmids, mainly IncI1α, IncFII, Col440I, IncHI2, IncN, and IncI2 (Delta) (Figure 3).

### Virulence Genes in *Salmonella* Telelkebir Population

The virulence gene profile for the 121 strains was carried out using the virulence factor database (VFDB) (Supplementary Table 1). The FJ001 strain harbored cytolethal distending toxin (*cdtB*) gene and carried fimbrial adherence genes and secretion system genes. A total of 55.81% of the genes (72/129) were conserved among all strains. Twenty-one genes were not present in any of the strains, and the rest of the genes were variable. All the strains carried fimbrial adherence genes *fim*, *csg*, and *inv*, the secretion system genes, *sopA*, *sopB*, *sopE2*, *spaOPQRS*, *spiC, sscAB, sifA*, and *pipB*, and the operons *sse* and *ssa*. The secretion system genes *pipB2, sopD2*, and *slrP*, and the effector genes, *sseK1, and sseK2*, and bacteriophage-related genes *sodCI, sspH2*, and *sspH1* genes were variable among the strains. Importantly, all strains (100%) were positive for the cytolethal distending toxin (CDT) genes (*cdtB, pltA*, and *pltB*). The *cdtB* gene is considered as one of the typhoid toxins of *Salmonella* Typhi, which causes cell arrest due to DNA damage (Chang et al., 2019). The BLASTp analysis of the proteins CdtB, PltA, and PltB of *Salmonella* Telelkebir showed that all the proteins were highly similar (100–97%) to the CdtB, PltA, and PltB proteins of the available *S. enterica* strains in NCBI.

### Phylogenetic Analysis of *Salmonella* Telelkebir Population

To determine the phylogenetic relationship, Snippy v.4.4.4 was used to obtain SNP alignment. A total of 18 ST types were identified in 121 isolates from 10 countries across four continents (Figure 3 and Supplementary Table 1). The present study showed that ST450 is the predominant sequence type (33%, 40/121), followed by ST2222 (19%, 23/121), and ST2651 (9%, 11/121) (Figure 3). The FJ001 strain belongs to ST5494, and it clustered with a human isolate isolated from France. The R17.4518 obtained from Taiwan, China, had the ST2651 and clustered with the UK isolates. Interestingly, the two Chinese strains did not cluster together. Among the ST450 isolates, 29 isolates (72.5%) were obtained from humans; four isolates were recovered from animals (10%), whereas the source of the remaining seven isolates could not be obtained. The ST2222 and ST2651 strains were mainly isolated from humans (23 and 11 strains, respectively). The ST450 and ST2249 were associated with strains of animal origin. The phylogenomic analysis reveals that the closely related strains are from very diverse geographical origins. In the ST profiles, the UK region presented a substantial higher diversity among the examined isolates (Figure 3 and Supplementary Table 1). ST2155 was associated with the food products (baklava from Turkey).

### Core Genome Multilocus Sequence Typing Analysis of *Salmonella* Telelkebir Population

**FIGURE 4:**
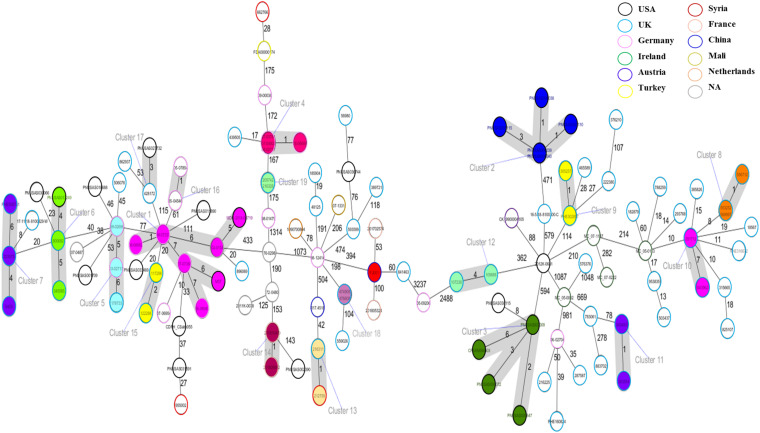
Minimum spanning tree of 121 *Salmonella* Telelkebir isolates from different origins are depicted here. The numbers on the connecting lines illustrate the numbers of different alleles between connected samples. Closely related samples are represented with shaded circles of closely related genotypes (≤7 different cgMLST alleles) are shaded with gray. The FJ001 strain is shaded in red.

The cgMLST based approach was employed to evaluate the genomic epidemiological features of the global *S.* Telelkebir strains. The cgMLST study was performed on a collection of loci that were shared by all *Salmonella* isolates, which were then used for gene by gene comparison. The difference in the nucleotide sequences of these loci determines the clustering of isolates (Tang et al., 2017; Pearce et al., 2018). According to cgMLST results, these samples were diverse as depicted by the different number of alleles between the isolates (Figure 4). Nineteen clusters and 68 singletons were identified in the global phylogenetic tree. The FJ001 strain did not form any cluster. The nearest neighbor for the FJ001 strain was 201702574, which were obtained from France with an allelic difference of 53 alleles. The clusters were perfectly coherent with the STs assignment. The ST450 group was subdivided into seven different clusters, with ST450-cluster (C1) being the dominant type. The ST450 strains were isolated from various geographical locations and different years and the reservoirs for these strains where humans (*n* = 4) and animals (*n* = 4) (Supplementary Table 1), whereas the cluster C1 mainly included the strains recovered from humans and animals that had an allelic difference of one to six alleles. Two *S.* Telelkebir isolates from China had resistant genes [*fosA7* and *aac(6′)-Iaa*] but belonged to different types both by MLST and cgMLST. The results suggested that the antimicrobial resistance gene profile varied according to the cgMLST clusters (Supplementary Table 1). The fosfomycin resistance genes were observed in C1, C4, C5, C6, C7, C13, C14, C15, C16, C18, and C19 clusters. The *qnrB19* resistance gene was present among clusters C1 (28.57%, 2/7), C8 (100%, 3/3), and C10 (100%, 2/2) and it was mainly associated with plasmid Col440I.

## Discussion

There are earlier reports on the isolation of uncommon *Salmonella* Telelkebir serovar (Tankson et al., 2006; Octavia et al., 2019). However, little is known about its resistance profile, epidemiology, and disease-causing potential. Previous reports suggested that *Salmonella* serotype Telelkebir is an infrequently reported variant that is mainly associated with exotic animal species, mostly reptiles (Berendes et al., 2007). Between 1995 and 2007, *S.* Telelkebir strains were obtained from animal-feed ingredients in Poland (Dera-Tomaszewska, 2012). Berendes et al. (2007) reported a case study where a 17-year-old girl had sepsis splenic abscesses caused by *Salmonella* serovar Telelkebir. The same variant was cultivated from the feces of the reptile pets that were held in the home of the patient. Our study demonstrates that the four global animal isolates had between one and six cgMLST allelic differences to the closest human isolates. In a previous study, three 2017 Bavarian *S.* Agona feed-origin outbreak strains were compared with a French outbreak isolate and 48 *S.* Agona isolates collected from 1993 to 2018, out of which 28 were epidemiologically outbreak related. The cgMLST analysis revealed that four most relevant clusters comprised 3 to 15 samples with a maximum within-cluster difference of zero to five alleles (Dangel et al., 2019). The study revealed that cgMLST can be used for reasonable, reproducible, and reliable high-resolution classification to monitor outbreak clusters and relationships among past or international cases, which could also be interpreted using representative public data.

Even though the FJ001 strain was pan-susceptible toward the tested antimicrobial agents, treatment with cefoperazone sulbactam alone or combined with levofloxacin failed when administrated to the patient. Interestingly, the WGS analysis revealed that the strain FJ001 had no antimicrobial resistance genes against quinolones and β-lactam class of antibiotics. One of the possible reason could be that for *Salmonella* spp. and *Shigella* spp., aminoglycosides, first- and second-generation cephalosporins and cephamycins may appear active *in vitro*, but are not effective clinically (CLSI, 2016). Also, according to “the 90–60 rule” coined by Rex and Pfaller (2002), a susceptible result in *in vitro* is associated with a favorable therapeutic response in 90–95% of patients. Various host or pathogen factors influence the efficiency of antibiotic therapy *in vivo*, such as the host immune system, site of infection (penetration of antimicrobial agents into the site), and bacterial virulence factors that may increase or hinder the immune response leading to poor clinical outcomes (Stratton, 2006; Ersoy et al., 2017). This could be the possible explanation of the ineffectiveness of these drugs in the patient.

The *fosA7* is a new antimicrobial resistance gene against fosfomycin that was recently identified in *S*. Heidelberg from broiler chickens in Canada (Rehman et al., 2017). The gene *fosA7* confers resistance to broad-spectrum antibiotic fosfomycin, which is extensively used to treat drug-resistant Gram-negative bacteria (Balbin et al., 2020). A total of 58.67% of the tested *S.* Telelkebir strains (71 strains) harbored the *fosA7* gene. The fluoroquinolones are the drugs of choice for the treatment of iNTS due to their broad-spectrum antimicrobial activity. Nevertheless, the extensive use of these drugs has led to the appearance of resistant strains globally, mainly in Gram-negative bacterial species. The *qnrB19* gene is one the most frequent variants of *qnr* genes globally, and out of the 121 strains, 12 isolates are positive for *qnrB19* gene (Supplementary Table 1; Ciesielczuk et al., 2013). The *qnr* is often found in association with genes that impart resistance to other antibiotics classes, e.g., β-lactams and aminoglycosides (Robicsek et al., 2006). In the present study, the *qnr* genes were mostly associated with aminoglycosides which suggests that *Enterobacteriaceae* strains harboring *qnr* resistance may denote a serious threat to public health. Also, the presence of *bla*_TEM__–1B_ gene was observed in three human *S*. Telelkebir isolates from the United Kingdom, Ireland, and Mali. The broad-spectrum β-lactamase enzymes can hydrolyze almost all β-lactams and are commonly linked with genes conferring resistance to several other classes of antibiotics (Bush and Bradford, 2016).

The screening of the virulence gene profile demonstrated that all *S*. Telelkebir isolates harbored various fimbrial genes (*bcf*, *fim*, *inv*, and *csg*) and secretion systems involved in cell invasion and bacterial viability in phagocytes. Previous results suggest that fimbriae are involved in differential intestinal colonization of animal species (Weening et al., 2005; Yue et al., 2012). The cytolethal distending toxin (CDT) or typhoid toxin is a bacterial genotoxin, which are encoded by several Gram-negative bacteria, including *S. enterica*. Our comparative genome analysis, for the first time, revealed that FJ001 strain and all the *Salmonella* Telelkebir strains harbored the gene *cdtB, pltA*, and *pltB* typhoid toxin gene, which highlights particular concern in public health. Typhoid toxin is recognized as a major virulence factor of *S*. Typhi, and it is reported to play a central role in the pathogenicity of *S*. Typhi. It has been observed that typhoid toxin is involved in the establishment of *S*. Typhi persistent infection most likely by altering the immune cell functions to its favor (Song et al., 2010; Chong et al., 2017). The genes encoding *Salmonella*-CDT (i.e., genes *pltA*, *pltB*, and *cdtB*) have been characterized in around 40 NTS serovars (den Bakker et al., 2011; Mezal et al., 2014a). Previous reports suggest that the amino acid alignments of CdtB, PltA, and PltB are extremely conserved among *S*. *enterica* serotypes (Rodriguez-Rivera et al., 2015). The BLASTp analysis of all the proteins CdtB, PltA, and PltB of *Salmonella* Telelkebir strains showed high sequence similarity (100–97%) to *S. enterica* strains. This suggests that the CdtB, PltA, and PltB are highly conserved among *S*. *enterica* serotypes. Also, cellular-level *Salmonella* CDT significantly alters the outcome of infection by inducing DNA damage, which is associated with a cell cycle arrest and activation of the host cell’s DNA damage response (Chang et al., 2019). Earlier findings revealed that *S*. Javiana isolates harboring *cdtB*, *pltA*, and *pltB* caused cytoplasmic and nuclear enlargement together with cell cycle arrest in G2/M phase, increased invasion and cytotoxicity toward HeLa cells that are characteristic of CDT activity (Mezal et al., 2014a). Recently, Xu et al. (2020) reported the presence of the *cdtB, pltA*, *and pltB* genes in iNTS serovar *S*. Uzaramo, which showed a higher killing rate than the classic gastrointestinal infectious agent *S*. Typhimurium and suggested it to be a possible explanation of the invasiveness of these serovars. Ahn et al. (2021) demonstrated that even when host cells infected with *S*. Typhi are treated with antibiotics, typhoid toxin is continuously secreted by antibiotic-resistant *S*. Typhi. These NTS serovars might have acquired these unique virulence factors through horizontal gene transfer during homologous recombination between *S.* Typhi and non-typhoidal serovars, by a prophage that integrates into the bacterial DNA chromosome (Bakker et al., 2011), and it might have led to a higher capacity of invasion in NTS (Mezal et al., 2013; Mezal et al., 2014b).

The SNP-based phylogenomic analysis also revealed that the strains clustered together irrespective of the time of isolation, source, and geographical location. This could be the result of global dissemination, due to the convenient and regular global travels, there is an increase in the possibility for fast and extensive transmissions of bacterial pathogens, which permits these isolates to effortlessly cross geographical barriers. Regardless of the improved surveillance followed today in response to the outbreak of infectious diseases, transmission connections cannot be identified in the event of an infectious disease outbreak due to missed screening, antimicrobial therapy suppression, and difficulties recognizing contacts (Jia et al., 2019).

## Conclusion

In summary, we reported a bloodstream infection caused by an uncommon NTS serovar Telelkebir in mainland China. These infrequently reported *Salmonella* serovars can possibly be misidentified in clinics, and the actual threat possessed by these serovars may be underestimated. Although the real threat of human diseases caused by this infrequently reported *Salmonella* serovar remains unknown, the combination of unique virulence factors and antimicrobial resistance genes carried by these minority NTS may lead to adverse clinical outcomes, demonstrating the necessity for an enhanced surveillance for the clinically important typhoid-toxin-containing serovars.

## Data Availability Statement

The datasets presented in this study can be found in online repositories. The names of the repository/repositories and accession number(s) can be found below: https://www.ncbi.nlm.nih.gov/, PRJNA666303.

## Author Contributions

YQ, RN, XX, and MY conceptualized the study, performed the data curation, and formulated the methodology. YQ, RN, and XX were in charge of the resources and performed the investigation. YQ, RN, XX, SW, and HP performed the formal analysis and validation. MY was in charge of the software and visualization. KZ and MY supervised the study, were in charge of project administration, and acquisition of the funding. RN wrote the original draft of the study. RN and MY reviewed and edited the final manuscript. All authors contributed to the article and approved the submitted version.

## Conflict of Interest

The authors declare that the research was conducted in the absence of any commercial or financial relationships that could be construed as a potential conflict of interest.

## Publisher’s Note

All claims expressed in this article are solely those of the authors and do not necessarily represent those of their affiliated organizations, or those of the publisher, the editors and the reviewers. Any product that may be evaluated in this article, or claim that may be made by its manufacturer, is not guaranteed or endorsed by the publisher.

## Footnotes

1
http://enterobase.warwick.ac.uk

2
https://www.ncbi.nlm.nih.gov/sra

3
http://rast.nmpdr.org/

4
https://blast.ncbi.nlm.nih.gov/Blast.cgi?PROGRAM=blastp&PAGE_TYPE=BlastSearch&LINK_LOC=blasthome

5
https://cge.cbs.dtu.dk/services/PlasmidFinder/

6
https://cge.cbs.dtu.dk/services/ResFinder/

7
https://lfz.corefacility.ca/sistr-app/

8
https://cge.cbs.dtu.dk/services/SeqSero/

9
https://cge.cbs.dtu.dk/services/MLST/

## Supplementary Material

Supplementary Table 1 A complete analytic information for 121 the examined *Salmonella* Telelkebir isolates in this study.

Supplementary Table 2 A summary table of the genomic assembly information for 121 the examined *Salmonella* Telelkebir isolates.

Supplementary Table 3 cgMLST distance matrix table with a pairwise comparison of allelic differences among each two of 121 *S*. Telelkebir isolates.

Supplementary Table 4 Minimum inhibitory concentration values for *S*. Telelkebir strain FJ001 under various different conditions.
